# Botanical Report

**Published:** 1811-04

**Authors:** 


### BOTANICAL REPORT.

SINCE our last Report two numbers of the BOTANICAL MAGAZINE have appeared, the contents of which we shall proceed to enumerate, and occasionally to comment upon.

Aloë *foliolosa*. Under this article Mr. Ker has given a new generic character of the genus ALOË; the principal additional character in which appears to us to be its having winged seeds. This species is nearly allied to A. *spiralis*, from which, and from the cushion-aloes, it differs remarkably in habit from the lengthening of the caudex, still closely covered with the foliage.

Aloë *recurva*, *venosa* of Lamarck, and Aloë *mirabilis*, both belong to that division of the genus which bear a bilabiate corolla with the laciniæ rolled back.

Aloë *virens*. This is one of Mr. Haworth's species which is not taken up in the Hortus Kewensis, and was not before mentioned by any author: it has long scarlet reflexed flowers.

Aloë *margaritifera*, γ. *minima*. *Minor* has been before published under the name, however, of *media*.

Aloë *arachnoides*, β. *pumila*; the *atrovirens* of Decandolle.

Aloë *mitræformis*. β. *brevifolia*. This plant is considered as a distinct species by Haworth, but the *brevifolia* of the new edition of the Hortus Kewensis and of Decandolle is a different plant; the *prolifera* of Haworth. Mr. Ker has remarked that the synonym quoted from Decandolle in the Hortus Kewensis belongs to α, and not to β, to which it is there erroneously referred. We are glad to see that this ingenious botanist continues to illustrate this difficult genus, the more particularly, as, from the succulent nature of these plants, very little satisfaction can be obtained from consulting different herbariums in regulating the synonyms. It is especially satisfactory too, that so many of the drawings are made from Mr. Haworth's collection, because this gentleman's monograph of this genus in the 7th volume of the Linnæan Transactions being the most complete of any we have, these figures serve to identify his species with certainty, as being taken from the same plants as he has described; and thus give an additional value to his work.

Strumaria *crispa*, the Amaryllis *crispa* of Jaquin and other authors. This article contains a new generic character, and an enumeration of what Mr. Ker considers as a species belonging to this species.

In Dr. Sims's department we find

Hibiscus *surattensis*, a beautiful and singular annual, of which we have not before any coloured figure, perhaps indeed no figure at all, for it is rather doubtful whether the other figures may not all belong to a different species. It is a pity that the flowers are expanded for so short a time.

Diosma

Diosma *pulchella*. This beautiful little shrub well merits its name, and, as Diosma is become so extensive a genus, we can but wish that Bergius's division at first adopted by Linnæus and afterwards given up, had been retained, and the name of Hartogia applied to all such as had three capsules, without regard to the separation of the sexes.

Justicia *nervosa*. The Eranthemum *pulchellum* of our gardens, where it has long been known as a very ornamental inhabitant of the stove, producing its fine violet-blue flowers through the greater part of the year. Dr. Sims remarks, that, as this plant was referred to Justicia with the trivial name of *pulchella* by Ker before the publication of Vahl's work, and the Hortus Kewensis to that of Vahl, who evidently was not aware of its being the Eranthemum *pulchellum*. Linnæus took up the genus Eranthemum from an imperfect specimen, not improbably of this very plant, in Herman's herbarium. Notwithstanding these reasons, however, Dr. Sims has continued Vahl's name, because it was adopted in the Hortus Kewensis, which he considers as being likely to become the standard for names of plants cultivated in our gardens. This is also the Ruellia *varians* of Ventenat's Hortus Celsii.

Nymphæa *nitida*. This is considered as a new species of Water-lily, having a near affinity with N. *odorata*, but not, like that, hardy enough to bear our winters, when planted in an open pond, but requiring the treatment of a tropical production. Its native country seems to be unknown.

Nymphæ *rubra* (var. β.) *rosea*. Native of the East Indies, and a very magnificent plant.

Lotus *australis*. Not unlike in habit to our Lotus *corniculatus*, but producing delicate crimson flowers.

Cnicus *spinosissimus*. By the help of the Linnæan herbarium, Dr. Smith has confirmed to Dr. Sims the truth of Haller's assertion, that Gmelin's Siberian plant is different from this, although Wildenow, and most other botanists, continue to quote the Flora Sibirica in the synonimy of this species.

English Botany, for the two last months, contains

Chenopodium *botryodes*. This is considered by Dr. Smith as a new species, first pointed out to him by the accurate Mr. Wigg, growing near Yarmouth. We should be inclined to think it may be a vaiety of Ch. *rubrum* for any thing Dr. Smith has told us here.

Ulmus *glabra*. We have often been puzzled to make out our elms in our walks. It appears that there are more species than we were aware of, and that they have not generally been understood. Mr. Forster, who has paid so much attention to English botany, is of opinion, that Dr. Smith's Ulmus campestris, t. 1886, is the *Ulmus minor folio angusto scabro* of Goodyer and Ray, and very little known out of Norfolk. And that his *suberosa*, t. 2161, is Ulmus vulgatissima folio lato scabro of those writers.

Meum *athamanticum*. Native of Scotland and the northern parts of England and Wales. There seems to have been much difficulty in determining to what genus to refer this plant. Linnæus in the Species Plantarum called it Athamanta *Meum*, in his Systema Vegetab.

getab. he removed it to Æthusa. Jacquin restored the old name, which has been since generally followed.

Potamogeton *gramineum*; about which there appears to have been some difficulty, as Lightfoot mistook the plant altogether.

Lychnis *alpina*, first discovered in this island by Mr. Geo. Don, on rocks near the summit of Clova mountains, in Angusshire.

Euphorbia *Lathyris*. Although we suspect that this plant owes its origin to our gardens, being found chiefly in the neighbourhood of towns, yet it establishes itself so perfectly in many places, that it may have as good a right to be considered as belonging to the English Flora as some others.

Centaurea *Isnardi*, from the island of Jersey. There is a species of Centaurea which appears to us to be distinct from *nigra*, though probably confounded with it, so common that we have found it among the straw in hackney-coaches. It has no blackness about it; has generally a nearly undivided stem; leaves very narrow, and terminating in a sort of petiolus; what is this?

Asplenium *viride*. Trichomanes ramosum of Linnæus and other authors is a variety of this.

Asplenium *alternifolium*, a sort of intermediate species between *septentrionale* and *Ruta muraria*; discovered in the south of Scotland by Mr. Dixon. It has been long known in Germany. We suspect it may be more frequent than is supposed in our island, as we recollect to have found specimens, which we were at a loss to determine whether to refer them to *septentrionale* or *Ruta muraria*.

Our country readers will be pleased to hear that the second volume of the Hortus Kewensis is printed, and will speedily be published. The continued publication of this useful and scientific work is not likely to be retarded by the death of Mr. Dryander.

